# Pediatric growing teratoma syndrome of the ovary: A case report and review of the literature

**DOI:** 10.1097/MD.0000000000022297

**Published:** 2020-09-18

**Authors:** Takanori Oyama, Takuo Noda, Kana Washio, Akira Shimada

**Affiliations:** a Department of Pediatric Surgery; b Department of Pediatrics, Okayama University Hospital, Okayama, Japan.

**Keywords:** growing teratoma syndrome, immature teratoma, ovarian tumor, pediatric

## Abstract

**Rationale:**

Growing teratoma syndrome is defined as an increase in tumor size during or after systemic chemotherapy for germ cell tumors. These cases involve normal tumor maker levels and histological features of only mature teratoma. We report a rare case of an ovarian immature teratoma in a Japanese child that was diagnosed as growing teratoma syndrome.

**Patient concerns:**

A 12-year-old girl presented a painful abdominal mass. She underwent left salpingo-oophorectomy for grade 1 immature teratoma in the left ovary. She did not undergo additional chemotherapy or radiotherapy. Four months later, she presented with grade 3 immature teratoma disseminated into the abdomen and pelvis. Chemotherapy resulted in the tumor maker levels returning to their normal ranges, although the tumors had grown slightly.

**Diagnosis:**

The specimens resected by laparotomy after the chemotherapy consisted of mature tissue predominantly, although primitive neuroepithelium was observed in a small part of the specimen. The pathological diagnosis was grade 1 immature teratoma, notwithstanding the clinical diagnosis was growing teratoma syndrome based on the clinical features and pathogenesis.

**Interventions:**

Laparotomy was performed at 7 months after the first operation, with resection of various tumors as well as the rectum, sigmoid colon, residual left fallopian duct, and a small part of the ileum and omentum. Some small tumors at the parietal peritoneum were ablated, although many tiny tumors around the uterus were left untreated.

**Outcomes:**

The patient has been free from recurrence for 5 years.

**Lessons:**

Growing teratoma syndrome can develop in children, and their tumor size is comparable to that in adolescents and adults. Furthermore, development of growing teratoma syndrome from a primary germ cell tumor is presumably faster in children than in adolescents and adults. Complete resection of all growing teratoma tissue is recommended, although fertility-sparing surgery should be considered when possible.

## 1 Introduction

Growing teratoma syndrome (GTS) is defined as an increase in tumor size during or after systemic chemotherapy for germ cell tumors, with normal tumor markers and histological features of only mature teratoma. Logothetis et al^[1]^ first used this term in 1982, although DiSaia et al^[2]^ previously described a similar phenomenon as “chemotherapeutic retroconversion” in 1977. Amsalem et al^[3]^ subsequently concluded that GTS and chemotherapeutic retroconversion were probably the same phenomenon.

Although GTS is well known in male patients with germ cell tumors, ovarian GTS is less commonly reported. According to Li et al,^[4]^ who reviewed 101 cases reported in the English literature, ovarian GTS typically occurs in adolescents and young adults (median age at diagnosis of primary immature teratoma: 22 years), and only 8 cases (7.9%) were diagnosed in children. Thus, we report a rare case of an ovarian immature teratoma that was diagnosed as GTS in a 12-year-old Japanese girl, and review the literature regarding ovarian GTS in children.

## 2 Case report

A 12-year-old girl was admitted to a local hospital with a painful abdominal mass. Abdominal computed tomography (CT) revealed a large cystic mass containing calcified tissues and fat in the pelvic cavity. Serum tumor marker levels were normal (alfa-fetoprotein [AFP]: 11.25 ng/mL, beta-human chorionic gonadotropin [beta-HCG]: <0.10 mg/mL), although serum CA125 levels were not measured. Left salpingo-oophorectomy was performed based on a suspicion of a teratoma in the left ovary. Interestingly, malignant cells were not observed in the ascites despite previous rupture of the tumor. Gross total resection was achieved and the pathological diagnosis was grade 1 immature teratoma. No additional chemotherapy or radiotherapy was performed.

**Figure 1:**
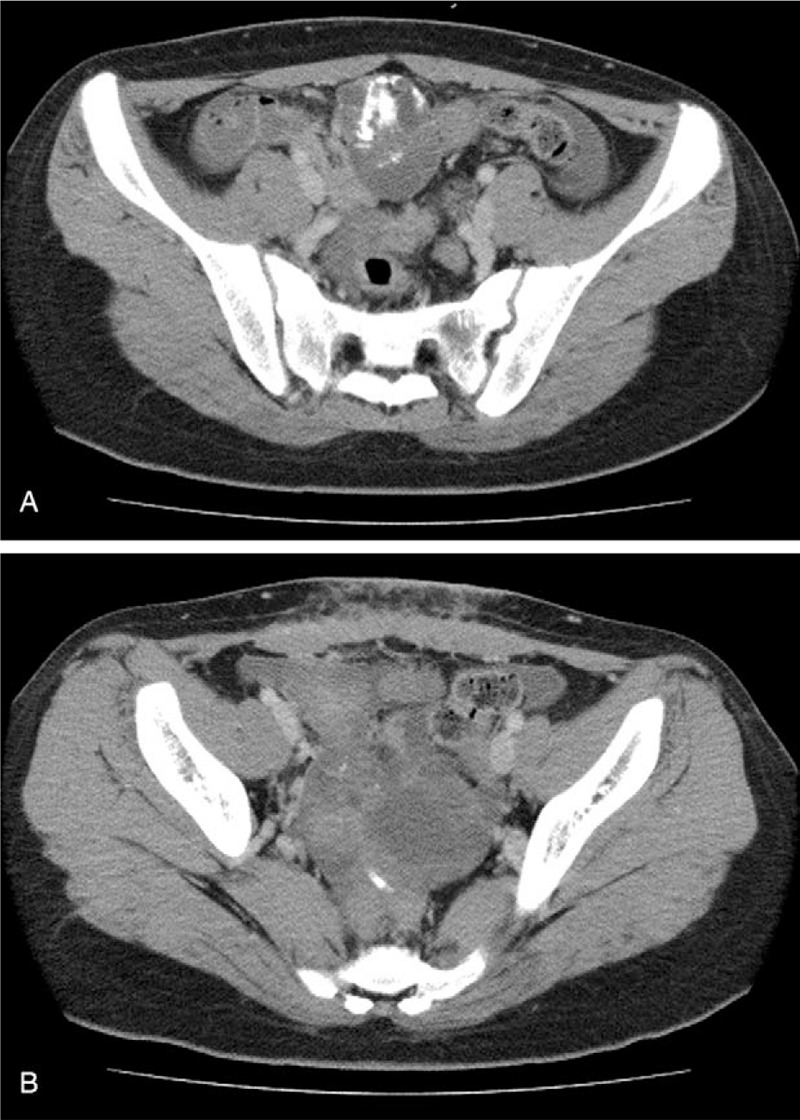
Abdominal computed tomography findings from 4 months after the first operation revealed multiple multinodular tumors containing calcified and fatty tissues (A). Invasion or lymph node swelling were not detected. Small amounts of ascites were observed around the liver and in the pelvic cavity (B).

**Figure 2:**
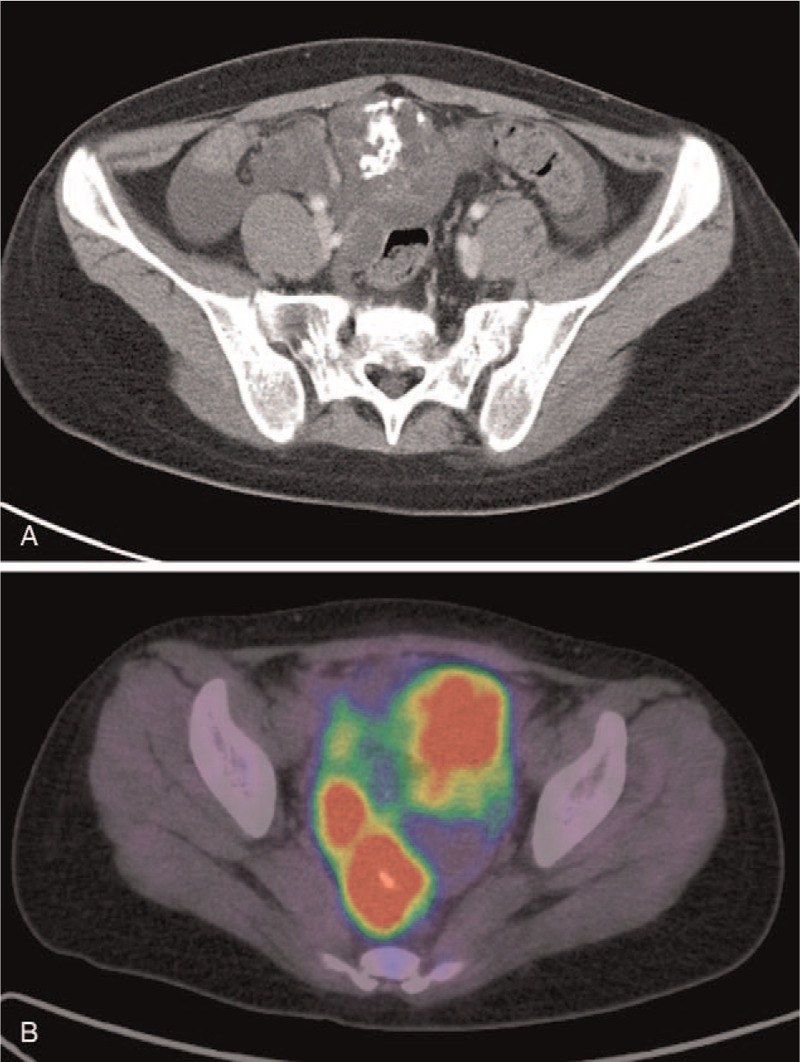
Post-chemotherapy findings from abdominal computed tomography (A) and positron emission tomography–computed tomography (B). The tumor had grown (A), and abnormal accumulation was observed (B).

Four months after the operation, abdominal CT revealed multiple multinodular tumors with a maximum diameter of 9.0 cm in the pelvis, which contained calcified and fatty tissue (Fig. 1), although the lymph nodes were not enlarged. Open biopsy was used to obtain a specimen for histological evaluation, which supported a diagnosis of grade 3 immature teratoma with substantial primitive neuroepithelium. The patient was admitted to our hospital soon after the recurrence with lower abdominal distention and tenderness, and the elastic hard tumor was palpable. Serum tumor maker testing revealed that her CA125 level was elevated to 198.2 U/mL, but with normal levels of AFP (5.1 ng/mL) and beta-HCG (<0.40 mIU/mL), despite the tumor's immaturity. Treatment was started using 1 cycle of bleomycin, etoposide, and cisplatin (BEP) chemotherapy, which normalized the CA125 levels, but the tumor became slightly enlarged. The treatment was then changed to 1 cycle of ifosfamide, carboplatin, and etoposide (ICE) chemotherapy, although the tumor became further enlarged. After the ICE chemotherapy, positron emission tomography-computed tomography (PET-CT) revealed abnormal accumulation in the lower abdomen and pelvis, which was considered indicative of a recurrent lesion (Fig. 2).

**Figure 3:**
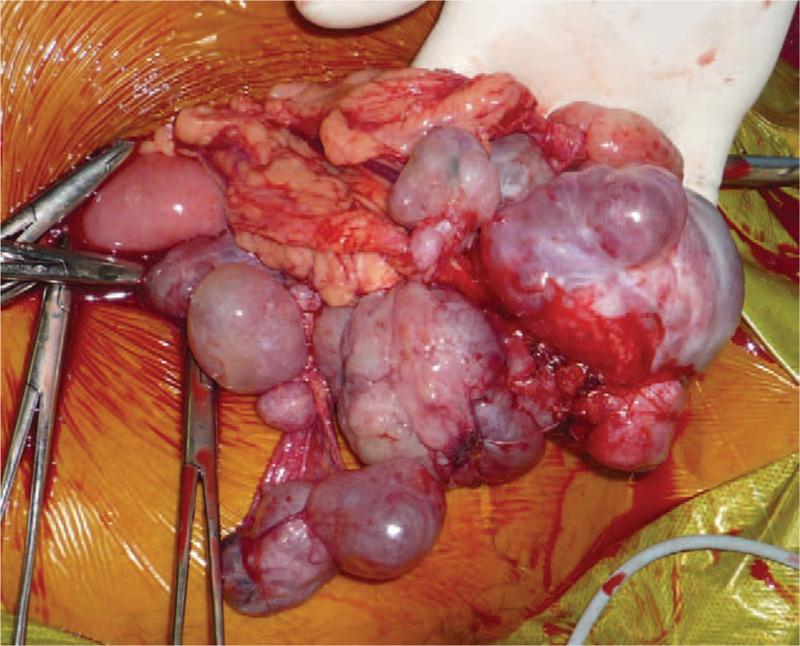
The surgery revealed numerous disseminated tumors in the great omentum.

Given the tumor growth, despite normalization of tumor markers after 2 chemotherapy cycles, we suspected GTS, which requires a pathological diagnosis and total resection even in asymptomatic cases. Thus, at 7 months after the first operation, we performed laparotomy (Fig. 3) and observed numerous disseminated tumors at the parietal and visceral peritoneum in the lower abdomen and pelvis. We resected the rectum and sigmoid colon because several disseminated tumors were firmly adherent to the ventral side of the rectum. We also resected the residual left fallopian duct and small parts of the ileum and omentum with the tumors. Some small tumors at the parietal peritoneum were ablated, although many tiny tumors around the uterus were not treated. The abdominal lymph nodes were not enlarged and the cytology results for the ascites were negative.

**Figure 4:**
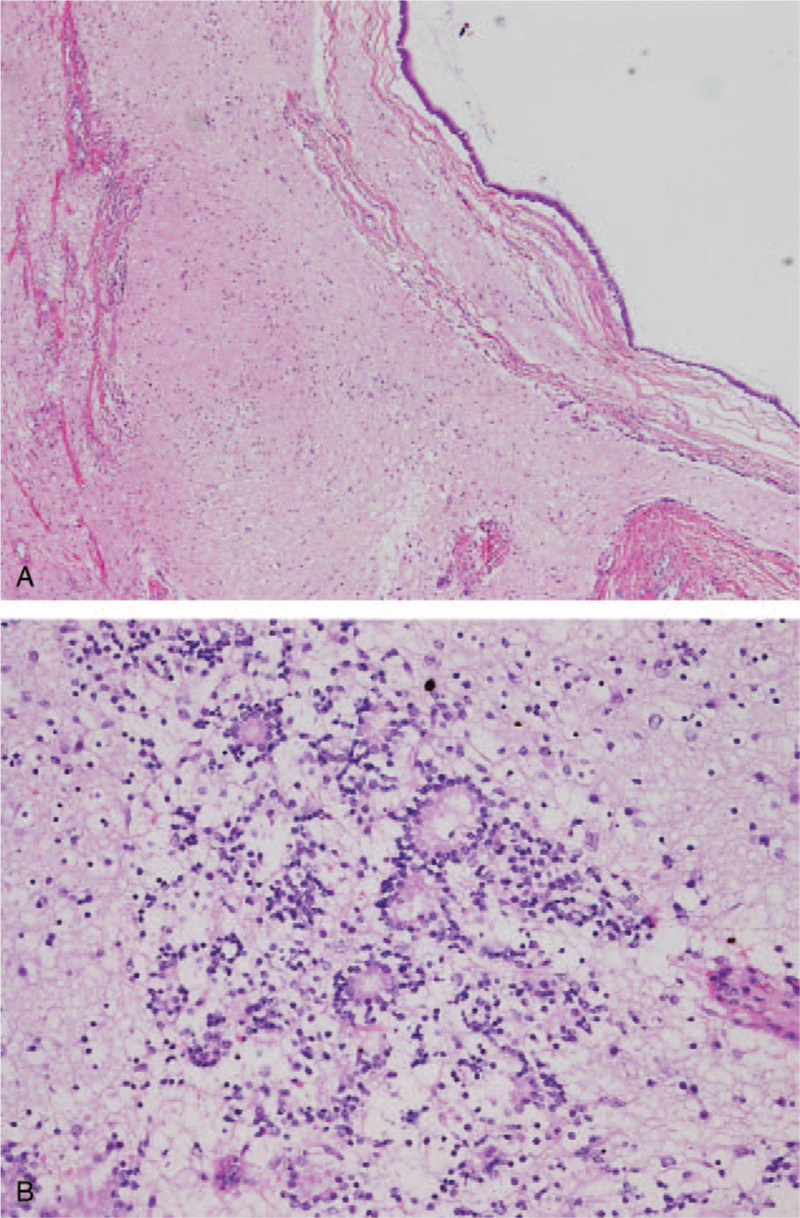
Pathological findings revealed mature tissue in almost all parts of the tumor (A), but a primitive neuroepithelium, represented by a slightly high cell density, in a small part of the tumor (B).

Pathological analysis of the resected specimens revealed that most of the specimen consisted of mature tissue, although primitive neuroepithelium was observed in a small part of the specimen (Fig. 4). The pathological diagnosis was grade 1 immature teratoma, which did not fulfill the pathological criteria for GTS (histological evidence of only mature teratoma). However, the pathogenesis agreed with that of GTS, and the use of additional chemotherapy could have converted the tumor into a mature teratoma. Thus, we considered the tumor to be GTS. The postoperative course was uneventful, and the patient was discharged 1 month after the operation with no additional chemotherapy or radiotherapy. The residual disseminated tumors have not enlarged during the 5-year follow-up.

## 3 Discussion

Ovarian immature teratoma is a relatively rare tumor that arises in young women, whose prognosis has been dramatically improved by BEP chemotherapy after fertility-preserving surgery. However, in the advanced cases with peritoneal metastasis, the prognosis remains poor even after appropriate treatment. In these cases, the diagnosis of GTS relies on 3 criteria:

(1) germ cell tumor growth during or after systemic chemotherapy,
(2) the normalization of previously elevated serum tumor markers, and
(3) the absence of immature components, other than mature teratoma, in the resected specimen.

In this context, ovarian teratoma of any grade can induce peritoneal implantation of mature glial tissues, which are known as gliomatosis peritonei (GP), and GTS is considered a type of GP, as both GTS and GP are associated with benign peritoneal glial implants regardless of the original tumor's malignancy grade.^[5]^

Two different pathogenic mechanisms have been proposed for GTS:

(1) chemotherapy induces the malignant differentiation of immature teratoma into a mature component^[2]^ or
(2) chemotherapy destroys only the immature malignant cells and spares the mature benign teratomatous elements.^[1]^

In the present case, the tumor had ruptured before the first operation, which might have been the cause of GTS development. Chemotherapy or radiotherapy was not performed after the first operation because there was no evidence of malignant ascites, dissemination was not detected using PET-CT, and the immature teratoma was histologically classified as grade 1. However, the disseminated tumor had enlarged at 4 months after the first surgery, and the histological classification was a grade 3 immature teratoma with an elevated CA125 level. Thus, the teratoma recurred as a higher-grade immature teratoma, rather than as GTS, which was induced by the BEP and ICE chemotherapy that was performed after the recurrence. Interestingly, the present case developed from a recurrent tumor, whereas the reported cases have developed from a primary tumor. In our case, the pathological diagnosis after the last surgery was grade 1 immature teratoma, and we suspect that further chemotherapy might have led to teratoma maturation that would ultimately have fulfilled the criteria for GTS. Therefore, we clinically diagnosed the tumor as GTS. Another important difference is that the only elevated tumor marker in the present case was CA125, while almost all reported cases have involved pre-treatment elevations of AFP and HCG, AFP and CA125, or only AFP.^[4]^ Thus, although the cause for this discrepancy is unclear, the tumor marker profile from present case appears to be rare.

The incidence of GTS in non-seminomatous germ cell tumors of the testis is 1.9% to 7.6%.^[1,6]^ In contrast, the incidence of ovarian GTS in ovarian immature teratomas is 12% to 19%,^[7,8]^ with the GTS originating from the right ovary in 57% of cases and the left ovary in 43% of cases. The median primary tumor size was reportedly 18.7 cm (range: 6–45 cm) and median subsequent tumor size was 8.6 cm (range: 1–25 cm). During the interval between the primary treatment and the diagnosis of ovarian GTS, the median tumor growth was 0.94 cm/month (range: 0.3–4.3 cm/month) and the median interval to diagnosis was 26.6 months (range: 1–264 months).^[4]^ While the GTS usually occurs at the primary site of the germ cell tumor, 13% of GTS cases involve metastatic sites.^[9]^

Ovarian GTS typically occurs in adolescents and young adults, with a median age of 22 years at the diagnosis of primary immature teratoma and only 7.9% of GTS cases being diagnosed during childhood.^[4]^ We have identified 17 cases of ovarian GTS in children in the English literature (Table 1).^[10–18]^ The youngest patient was 4 years old at the diagnosis of the primary germ cell tumor. The primary tumor size was 9 to 30 cm, which is comparable to in adolescent and adult patients, and our case involved the smallest reported primary tumor size (9 cm). The histology of the primary germ cell tumor was immature teratoma in 9 cases and mixed germ cell tumor in 5 cases. Eleven patients received chemotherapy, and the GTS diagnosis was made during the chemotherapy in 6 cases (including the present case), which suggests that GTS development is faster in children than in adolescents and adults. The GTS may cause obstruction or compression of abdominal structures, including the urinary tract, vascular structures, and gastrointestinal tract.^[11]^ Another report described mesenteric compression with bowel necrosis, renal failure due to ureteral compression, bowel obstruction, and bile duct obstruction.^[9]^ Fortunately, most children (including our patient) did not develop serious symptoms.

**Table 1:**
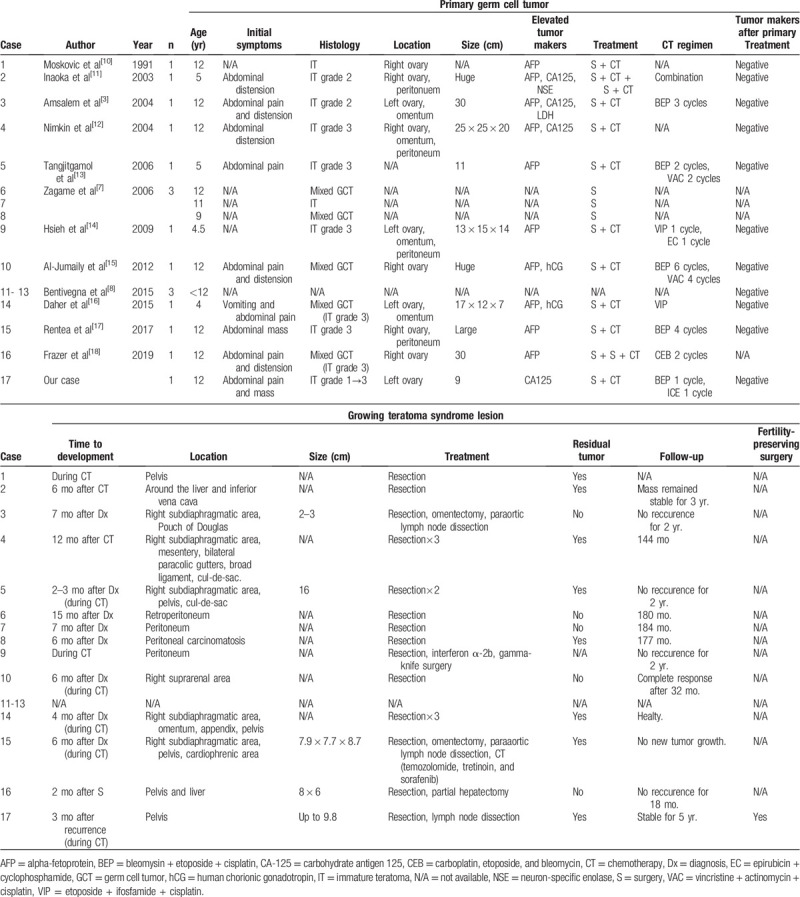
Ovarian growing teratoma syndrome in children: review of the literature.

The imaging-based diagnosis of GTS is supported by a mass with increasing size that contains fat, calcification, or cystic changes.^[19]^ Thus, similar to in ovarian cancer, PET-CT provides more information than CT alone, although a positive PET-CT finding does not always reflect malignancy.^[20]^ For example, positive PET-CT findings could be related to the high rate of glucose metabolism in brain tissues for cases of GTS (as in the present case).^[21]^

The GTS lesions are refractory to chemotherapy, which makes surgery the only curative treatment.^[19]^ Complete resection of all GST tissue is recommended, as incomplete resection can lead to progression or malignant transformation. For example, Shigeta et al reviewed 55 cases of ovarian GTS and reported that GTS recurred in 12.7% of cases (7/55) and underwent malignant transformation in 5.4% of cases (3/55),^[22]^ which can result in sarcoma, adenocarcinoma, or a primitive neuroectodermal tumor and carcinoid.^[23]^ Thus, if the residual teratoma components are caused by chemotherapeutic retroconversion, it appears logical that they would retain a high level of histopathological genetic aneuploidy and their malignant potential.^[24–26]^ Therefore, complete surgical resection is a highly desirable treatment,^[27]^ although recurrence or malignant transformation have not been reported in children. Nevertheless, fertility-sparing surgery is recommended when possible based on the findings of 2 studies. Li et al reviewed 101 patients with ovarian GTS and found that 5 of these patients became pregnant during the interval between the primary disease and GTS, with 1 patient becoming pregnant after the diagnosis of ovarian GTS. Bentivegna et al^[8]^ have also reported that 5 of 38 patients with ovarian GTS became pregnant after the diagnosis of ovarian GTS. Both studies mention that fertility-sparing surgery is recommended “when possible,” although the criteria for evaluating this possibility remain unclear. Further studies are needed to address this issue, as none of the reports regarding the children in Table 1 indicate whether they underwent fertility-sparing surgery, subsequently became pregnant, and relapsed.

The recurrence rate for ovarian GTS is up to 83% in cases with partial resection but only 0% to 4% in cases with complete resection.^[4]^ Among the reported children, only 2 cases involved complete resection (Table 1), and the median recurrence-free interval (from the final treatment for GTS to recurrence) was 24 months. These findings strongly indicate that a prolonged follow-up is needed in cases with GTS.^[22]^ Furthermore, several cases involved malignant transformation of GTS, which might be misdiagnosed as mature teratoma because the low-grade primitive neuroepithelium is only present in a small part of the specimen (as in the present case). Moreover, in the present case, we elected to leave many disseminated small immature tumors, and did not perform chemotherapy or radiotherapy because of the paucity of the primitive neuroepithelium, although close follow-up is necessary given the possibility of malignant transformation.

## 4 Conclusions

We encountered a rare case of an ovarian immature teratoma that was diagnosed as GTS in a Japanese child. In similar cases, the GTS can develop during early childhood and the tumor size is comparable to that in adolescents and adults, although the development of the GTS from the primary germ cell tumor is presumably faster in children. Complete resection of all GST tissue is recommended based on the possibility of malignant transformation, although fertility-sparing surgery should be considered when possible.

## Acknowledgment

The authors thank Editage for English language editing.

## Author contributions

**Conceptualization:** Takanori Oyama, Takuo Noda, Akira Shimada.

**Data curation:** Takanori Oyama.

**Investigation:** Takanori Oyama, Takuo Noda, Kana Washio, Akira Shimada.

**Project administration:** Takuo Noda, Akira Shimada.

**Supervision:** Takuo Noda.

**Visualization:** Takanori Oyama.

**Writing – original draft:** Takanori Oyama.

**Writing – review & editing:** Takanori Oyama, Takuo Noda, Kana Washio, Akira Shimada.

## Abbreviations

Abbreviations: AFP = alfa-fetoprotein, BEP = bleomycin, etoposide and cisplatin, Beta-HCG = beta-human chorionic gonadotropin, CA125 = carbohydrate antigen 125, CT = computed tomography, GP = gliomatosis peritonei, GTS = growing teratoma syndrome, ICE = ifosfamide, carboplatin, etoposide, NSGSTs = non-seminomatous germ cell tumors, PET-CT = positron emission tomography-computed tomography.
